# Proanthocyanidins from *Camellia kwangsiensis* with Potent Antioxidant and *α*-Glucosidase Inhibitory Activity

**DOI:** 10.3390/foods15030442

**Published:** 2026-01-26

**Authors:** Na Li, Qin Ni, Min Chen, Hong-Tao Zhu, Man Zhang, Takashi Tanaka, Ying-Jun Zhang

**Affiliations:** 1 State Key Laboratory of Phytochemistry and Plant Resources of West China, Kunming Institute of Botany, Chinese Academy of Sciences, Kunming 650201, China; lina1@mail.kib.ac.cn (N.L.);; 2 Yunnan Institute for Food and Drug Control, Kunming 650500, China; 3 State Key Laboratory of Phytochemistry & Natural Medicines, Kunming Institute of Botany, Chinese Academy of Sciences, Kunming 650201, China; 4 Graduate School of Biomedical Sciences, Nagasaki University, 1-14 Bunkyo-Machi, Nagasaki 852-8521, Japan; t-tanaka@nagasaki-u.ac.jp

**Keywords:** *Camellia kwangsiensis*, proanthocyanidins, antioxidant activity, *α*-glucosidase inhibitory activity, LC-MS identification, quantitative determination

## Abstract

This study aimed to systematically investigate the chemical constituents and bioactivities of the traditional wild tea plant *Camellia kwangsiensis* Chang. An HPLC method was first established to simultaneously quantify five major components. Subsequently, extensive isolation was performed using chromatographic techniques, and the structures of isolated compounds were elucidated by spectroscopic methods. Their biological potential was evaluated through antioxidant (DPPH and ABTS^+^ radical scavenging), *α*-glucosidase inhibitory, and anti-inflammatory (inhibition of nitric oxide production) assays. The LC-MS/MS analyses confirmed the absence of caffeine, theophylline, and theobromine. A total of 19 phenolic compounds were first isolated and identified, including one new proanthocyanidin, namely kwangsienin A (**1**), and 18 known phenolic components with six proanthocyanidins (**2**–**7**), one catechin (**8**), six flavonol glycosides (**9**–**14**), and five simple phenols (**15**–**19**). Notably, the proanthocyanidins displayed stronger or comparable antioxidant and *α*-glucosidase suppressive activity than the positive controls. In conclusion, *C. kwangsiensis*, rich in proanthocyanidins and naturally caffeine-free, represents a promising plant resource for developing decaffeinated functional tea beverages with antioxidant and hypoglycemic potential.

## 1. Introduction

Tea is among the oldest and most widely consumed beverages globally. It is typically made from the young, tender leaves and buds of two main varieties of the *Camellia* genus: *Camellia sinensis* (L.) O. Kuntze var. *sinensis* and *C. sinensis* var. *assamica* (Masters) Kitamura, both of which belong to the Theaceae family. In addition, other wild tea species belonging to the same genus, e.g., *C. taliensis* and *C. kwangsiensis*, have also been historically used for making tea by local people in their native regions [1].

Flavan-3-ols, such as catechins and proanthocyanidins, are the key constituents of both green tea and the original tea plants, known for their potent antioxidant, anti-inflammatory, antidiabetic, and antitumor effects [1,2,3,4,5]. Proanthocyanidins, commonly referred to as condensed tannins, represent a class of phenolic dimers and oligomers composed of catechin units [(epi)catechins, (epi)gallocatechins, (epi)afzelechins]. These compounds are found extensively in plants, and are further divided into several classes due to variations in hydroxylation patterns and the different linkages between their constitutive units [6,7]. Typically, they comprise no more than 1% of green tea, a level that is quite low compared to the content of flavan-3-ol monomers [8].

The health benefits of tea polyphenols have spurred interest beyond traditional beverages, including a significant growth in demand for decaffeinated tea. Conventional tea (*C. sinensis*) contains considerable caffeine (typically 20–40 mg/g), a substantial portion of which is extracted during brewing [9]. While beneficial for some, caffeine can cause adverse effects such as palpitations and insomnia in sensitive individuals, driving the preference for decaffeinated teas [10]. Consequently, recent research has focused on developing decaffeinated teas through cultivar selection, processing, and decaffeination technologies [11]. Furthermore, tea polyphenols hold potential for applications in nutraceuticals, cosmetics, medical applications, and food packaging materials [12,13].

Among various health attributes, the hypoglycemic effect of tea is of significance. A growing body of evidence indicates that tea consumption and its active compounds (e.g., polyphenols, polysaccharides) are inversely associated with the risk of diabetes through multiple mechanisms, such as inhibiting digestive enzymes, improving insulin sensitivity, and modulating gut microbiota [14]. Notably, the inhibition of carbohydrate-digesting enzymes like *α*-glucosidase is recognized as a primary mechanism for managing postprandial blood glucose levels. This underscores the importance of discovering tea resources with potent, relevant bioactivities.

The exploration of wild tea resources is pivotal for diversifying functional teas. China, particularly its southwest region, is the centre of origin for the genus *Camellia* section Thea, which encompasses a diverse array of species and varieties [1]. However, phytochemical and bioactivity research has largely been confined to the commercial cultivars, with only a handful of wild species (e.g., *C. taliensis*) receiving preliminary scientific attention. The systematic investigation of unexplored wild species is essential to unlock their unique phytochemical potential and value for sustainable utilization.

*C. kwangsiensis* Chang, featuring leathery, long oval leaves, white hairs on the apex buds and branches, and hairless leaves and petals, is an endemic tea species found in the broad-leaved forests of eastern Guangxi and southeastern Yunnan provinces in China, with an altitude of 1500–1900 m [1]. Its leaves are commonly utilized to make various types of tea (e.g., green tea, black tea), which are consumed by the local population. *C. kwangsiensis* remains an under-exploited wild resource with no commercial production at present. Thus far, no detailed phytochemical study of *C. kwangsiensis* has been reported. In this study, our initial chemical screening (as detailed in Section 3.1) of their tea leaves, collected from Tianlin, Guangxi Province of China, showed no detectable caffeine, theophylline, or theobromine. Instead, they were found to have a considerable amount of di-/oligomeric flavan-3-ols (i.e., proanthocyanidins) and flavonol glycosides. To scientifically assess their potential, this study was designed to systematically characterize the key phenolic components and evaluate their related bioactivities. This included establishing an HPLC method for quantifying five major components, isolating 19 phenolic compounds (one being new), and testing their antioxidant, *α*-glucosidase inhibitory, and anti-inflammatory effects.

## 2. Materials and Methods

### 2.1. General Procedure

UV spectra were obtained on a UV-2410PC ultraviolet-visible spectrometer (Shimadzu Co., Kyoto, Japan). A Thermo Nicolet iS10 series spectrometer was used to obtain IR spectra by KBr pellets (Thermo Fisher Scientific Inc., Waltham, MA, USA). The detection of optical rotations (OR) and circular dichroism (CD) spectra was carried out with a Rudolph Research Analytical Autopol IV automatic polarimeter (Rudolph Research Analytical, Hackettstown, NJ, USA) and an Applied Photophysics Chirascan V100 spectrometer (Applied Photophysics Ltd., Leatherhead, UK), respectively. The 1D and 2D NMR spectra were recorded in CD_3_OD using Bruker Ascend-600 and Ascend-800 spectrometers (Bruker Co., Karlsruhe, Germany). Chemical shifts (*δ*) are reported in parts per million (ppm), using Tetramethylsilane (TMS) (Bruker, Zurich, Switzerland) as an internal reference. Coupling constants are given in hertz (Hz). Electrospray ionization mass spectrometry (ESIMS) and high-resolution ESIMS (HRESIMS) analyses were measured on an Agilent 1290 UPLC/6540 Q-TOF spectrometer (Agilent Technologies Inc., Santa Clara, CA, USA). Ultrasonic extraction was carried out on a HU0260F series digital ultrasonic bath device (HENGAO T&D, Beijing, China). A FlexStation3 multifunctional microplate reader was used for absorbance reading (Molecular Devices Co., Sunnyvale, CA, USA).

### 2.2. Chemicals and Reagents

Column chromatography (CC) was carried out with Diaion HP20SS (75–150 μm, Mitsubishi Chemical Co., Ltd., Tokyo, Japan), MCI gel CHP20P (75–100 μm, Mitsubishi Chemical Co., Ltd., Tokyo, Japan), 25–100 μm Sephadex LH-20 (GE Healthcare Bio-Science AB, Uppsala, Sweden), 40–60 μm RP-18 (Merck, Darmstadt, Germany), and TSK gel Toyopearl HW-40F (37–70 μm, Tosoh Co., Ltd., Tokyo, Japan). Semi-preparative HPLC was performed on a Hanbon series HPLC system (Hanbon Sci. & Tech., Huai’an, China) with Agilent ZORBAX SB-C18 (5 μm, 250 mm × 9.4 mm) and Capcell Pak Phenyl (5 μm, 250 mm × 10 mm) columns, and the flow rate was 3 mL/min. Thin-layer chromatography (TLC) was performed on precoated silica gel GF254 plates (0.20–0.25 mm thickness, Qingdao Haiyang Chemical Co., Ltd., Qingdao, China), using various mobile phases consisting of benzene/ethyl formate/formic acid in ratios of 3:6:1, 2:7:1, and 1:7:1 (*v*/*v*/*v*). The spots were visualized by heating silica gel plates, which were sprayed with either ethanolic FeCl_3_ or anisaldehyde-H_2_SO_4_ reagent. Standards of proanthocyanidins B2, B5, and C1, (−)-epicatechin, and quercetin-3-*O*-*α*-L-rhamnopyranoside were obtained from Shanghai Macklin Biological Technology Co., Ltd. (Shanghai, China) and Sichuan Weikeqi Biological Technology Co., Ltd. (Chengdu, China), respectively.

### 2.3. Materials

*C. kwangsiensis* was collected from Tianlin county, Guangxi Province, China, and authenticated by Dr. Shi-Xiong Yang from Kunming Institute of Botany (KIB), Chinese Academy of Sciences (CAS). The voucher specimen (KIB-Z-20180965) is maintained at the State Key Laboratory of Phytochemistry and Plant Resources in West China, KIB, CAS.

### 2.4. Extraction Procedures

Two slightly different extraction protocols were used for different analytical purposes.

A. Extraction for LC-MS analysis:

The finely powdered plant material (1.500 g) was subjected to ultrasonic extraction (frequency: 28 kHz) with 70% aqueous methanol (MeOH, 100 mL) at room temperature for three 20 min intervals. The extract was subsequently filtered using a 0.22 μm nylon membrane to prepare it for LC-MS analyses.

B. Extraction for quantitative HPLC analysis:

For accurate quantification, a precisely weighted sample (6.000 g ± 0.300 g) was soaked overnight in 70% aq. MeOH (100 mL). Then, the solution was extracted with ultrasonic baths (frequency: 28 kHz) at room temperature for three cycles, each lasting 20 min. After filtration, the extract was adjusted to a final volume of 100 mL in a volumetric flask with 70% aq. MeOH and filtered again through a 0.22 μm nylon membrane before HPLC injection.

### 2.5. HPLC and LC-MS Method

HPLC profiles were conducted using an Agilent Zorbax SB-C18 column (4.6 × 150 mm, 5 μm), employing a gradient elution of 4–40% acetonitrile (MeCN) in water over 45 min. The column temperature was maintained at 30°C, with a flow rate of 1.0 mL/min and an injection volume of 5 μL. The Agilent 1290 Infinity II diode-array detector was set at four wavelengths of 203, 210, 254, and 280 nm. MS analysis was carried out using an ESI interface, operating in full-scan MS mode within the mass range of 100 to 1500 *m*/*z*. The sample was analyzed in both negative and positive ionization modes. Parameters for ESIMS included an ion spray voltage of 4 kV, and the capillary temperature was set at 300 °C.

### 2.6. Quantitative Determination

Sample analysis in triplicate was carried out on a Waters chromatograph, fitted with a 2695 Separation Module and a 2996 Photodiode Array Detector (Waters Co., Milford, CT, USA). Chromatographic partitioning was achieved using a Cosmosil 5C_18_-MS-II column (4.6 × 250 mm, 5 μm), with a gradient elution of 4–40% acetonitrile–water as mobile phase over 55 min. A 10 μL injection volume was used, and the flow rate was set to 1.0 mL/min. Detection was performed at a wavelength of 210 nm.

For the simultaneous determination of five components in the mixed standard solution and extracted samples, method validation was completed, focusing on linearity, recovery, and precision. Linearity was confirmed by calculating the coefficient of determination after constructing the calibration curves at different concentrations of 30 to 400 µg/mL, or 12 to 160 µg/mL. The recovery rate was assessed by spiking samples (around 1 times the contents) for five components. Precision tests for both intra- and inter-day variations were performed at two levels. The intra-day evaluation was determined by performing six injections of the standard solution of the tested compounds on the same day. For the inter-day evaluation, the same solution was determined successively over 6 d. The limit of detection (LOD) and limit of quantification (LOQ) were established according to signal-to-noise ratios (S/N) of 3:1 and 10:1, respectively (Figure S1, and Tables S2 and S3).

### 2.7. Extraction and Isolation

*C. kwangsiensis* (27 kg) was extracted with 60% acetone in water for four cycles (each lasting 7 d) at ambient temperature and filtered. Once the organic solvent was evaporated under reduced pressure, the combined filtrate was subjected to partitioning with ethyl acetate (EtOAc). The EtOAc layer (256 g) was first loaded onto a Diaion HP20SS column and eluted with a stepwise MeOH-H_2_O gradient (0:1 to 1:0, *v*/*v*), affording six primary fractions (Fr. 1–Fr. 6).

Fr. 2 (111 g) was subjected to CC over Sephadex LH-20 (MeOH-H_2_O, 0:1→1:0) to yield five subfractions (Fr. 2-1–Fr. 2-5). Of these, Fr. 2-2 (50 g) was further divided into four smaller fractions (Fr. 2-2-1–Fr. 2-2-4) using MCI-gel CHP20P CC (MeOH-H_2_O gradient). Fr. 2-2-1 yielded **3** (480 mg), **4** (10.0 mg), and **15** (10.3 mg) after sequential CC on Sephadex LH-20 and MCI-gel CHP20P, with a MeOH-H_2_O (0:1 to 1:0) gradient, and semi-preparative HPLC (MeCN-H_2_O). Similarly, compounds **1** (142 mg), **2** (200 mg), **5** (196 mg), **18** (3.8 mg), and **19** (4.2 mg) were isolated from Fr. 2-2-2 via repeated CC (Sephadex LH-20, MCI-gel CHP20P), followed by semi-preparative HPLC. From Fr. 2-2-3, compounds **9** (2.8 g), **10** (169 mg), **11** (30 mg), **14** (2.2 mg), and **16** (7.2 mg) were isolated using TSK gel Toyopearl HW-40F and MCI-gel CHP20P.

Fr. 3 (70 g) was fractionated by MCI-gel CHP20P CC (MeOH-H_2_O gradient) to afford four subdivisions (Fr. 3-1–Fr. 3-4). Compound **7** (512 mg) was separated from Fr. 3-2 (5 g) by successive CC on Sephadex LH-20 and TSK gel Toyopearl HW-40F. Fr. 3-4 (21 g) was further subjected to Diaion HP20SS (MeOH-H_2_O gradient) to yield eight subfractions (Fr. 3-4-1–Fr. 3-4-8). From these, compound **8** (350 mg) was purified from Fr. 3-4-3 (500 mg) via Sephadex LH-20 CC, while repeated CC (Sephadex LH-20, TSK gel Toyopearl HW-40F, and RP-18) and semi-preparative HPLC of Fr. 3-4-6 (7 g) resulted in the isolation of **6** (5.4 mg), **12** (100 mg), **13** (40 mg), and **17** (1.6 mg).

### 2.8. Compound ***1***

Orange amorphous powder; ESI-MS: *m*/*z* 865 [M − H]^−^, HRESI-MS: *m*/*z* 865.1980 [M − H]^−^ (calcd for C_45_H_37_O_18_, 865.1985); ${\left[\alpha \right]}_{D}^{21}$−21.59 (*c* 0.12, MeOH); UV *λ*_max_ (methanol) (log *ε*): 282 (4.21), 203 (5.28) nm; IR (KBr): *ν*_max_ 3422, 1612, 1520, 1446, 1284, and 1105 cm^−1^ for the ^1^H and ^13^C NMR data (see Table 1, Figures S3–S12).

### 2.9. Thiol Degradation

The trimer **1** (30 mg), dissolved in 70% ethanol (EtOH) (0.5 mL), was cleaved by reacting with a mixture of 1.5 mL of 5% mercaptoethanol in 60% ethanol with 0.1% hydrogen chloride (HCl), and the reaction was heated at 70 °C for 7 h. The resulting mixture was then separated on Sephadex LH-20, using a stepwise MeOH-H_2_O gradient (0–100%, 5% increments) as the eluent. This process yielded three products, and their 1D NMR data are provided in Table S1.

### 2.10. Antioxidant Assay

The antioxidant activities of the isolates were assessed using the 2,2-diphenyl-1-picrylhydrazyl (DPPH) and 2,2′-azino-bis(3-ethylbenzothiazoline-6-sulfonic acid) (ABTS^+^) radical scavenging assays, with ascorbic acid and 6-hydroxy-2,5,7,8-tetramethylchroman-2-carboxylic acid (Trolox) served as the respective positive controls.

DPPH assay: Briefly, 100 µL of a methanolic sample solution at various concentrations (2–1000 µg/mL) was mixed with 100 µL of a 100 µM DPPH methanol solution in a 96-well plate. The reaction was incubated in the dark at room temperature for 15 min, after which the absorbance was measured at 490 nm.

ABTS^+^ assay: The ABTS^+^ radical cation was generated by reacting 7 mM ABTS with 2.45 mM potassium persulfate and storing the mixture in the dark at room temperature for 12–16 h. The stock solution was diluted with methanol to an absorbance of 0.70 ± 0.02 at 734 nm to obtain the working solution. For the assay, 10 µL of sample was reacted with 200 µL of ABTS^+^ working solution for 6–8 min at room temperature, and the absorbance was immediately read at 405 nm.

For both assays, the radical scavenging activity was calculated as follows: Scavenging activity (%) = [(A_control_ − A_sample_)/A_control_] × 100. Detailed procedures can be found in our previous publication [15].

### 2.11. α-Glucosidase Inhibitory Assay

As recorded in our previous article [16], the *α*-glucosidase inhibitory activity of isolates was conducted with a model for screening enzyme-inhibitors, employing 4-nitrophenol-*α*-D-glucopyranoside (PNPG) as the substrate. Acarbose and quercetin were the positive controls. The test compounds were dissolved in dimethyl sulfoxide (DMSO) and serially diluted with phosphate buffer (0.1 M, pH 6.9) to final concentrations ranging from 0 to 200 µM. The reaction mixture in each well contained 50 µL of sample solution and 100 µL of *α*-glucosidase solution (0.025 U/mL in buffer) and was preincubated at 37°C for 10 min. The reaction was initiated by adding 50 µL of PNPG substrate solution (final concentration 1 mM), followed by incubation at 37°C for 50 min. The enzymatic hydrolysis was terminated by adding 50 µL of 0.2 M Na_2_CO_3_ solution. The amount of released *p*-nitrophenol was quantified by measuring the absorbance at 405 nm using a microplate reader. The inhibition percentage was calculated as [(A_control_ − A_sample_)/A_control_] × 100%.

### 2.12. Anti-Inflammatory Assay

The anti-inflammatory activity of isolates was evaluated based on their ability to inhibit nitric oxide (NO) production in lipopolysaccharide (LPS)-stimulated RAW264.7 macrophage cells, and the specific method was recorded in our previous report [17]. L-NMMA, a widely recognized inhibitor of NO synthase (NOS), served as the positive control. Murine macrophage cell line RAW264.7, purchased from the Cell Bank of the Chinese Academy of Sciences (Shanghai, China), was seeded into 96-well plates and induced by 1 μg/mL LPS. Dissolved in DMSO, the test compounds were then introduced into the cell culture medium to make a final concentration of 50 μM. After the cells were cultured overnight, the absorbance of NO production in the supernatant of the medium was measured at 570 nm. Cell viability in RAW264.7 was detected by adding 4-[5-[3-(carboxymethoxy)phenyl]-3-(4,5-dimethyl-1,3-thiazol-2-yl)tetrazol-3-ium-2-yl]benzene-sulfonate (MTS reagent) to the remaining medium, allowing for the exclusion of potential cytotoxic effects from the test compounds. The NO production inhibition percentage was calculated using the following formula: % inhibition = (E − S)/E × 100 (E is the absorbance of the non-drug treatment group, and S is the absorbance of the sample group).

### 2.13. Data Presentation

All bioassays (DPPH, ABTS^+^, *α*-glucosidase inhibition, and anti-inflammation) were performed with three independent replicates (n = 3). Data are expressed as the mean ± standard deviation (SD). The half-scavenging concentration (SC_50_) and half-maximal inhibitory concentration (IC_50_) values were calculated by non-linear (or linear) regression analysis of the dose–response curves. The analysis was carried out using GraphPad Prism (version 10.1.2). Biological activities of the tested compounds are discussed based on the direct comparison of these derived values (mean ± SD).

## 3. Results and Discussion

### 3.1. HPLC and LC-MS Analysis

**Figure 1:**
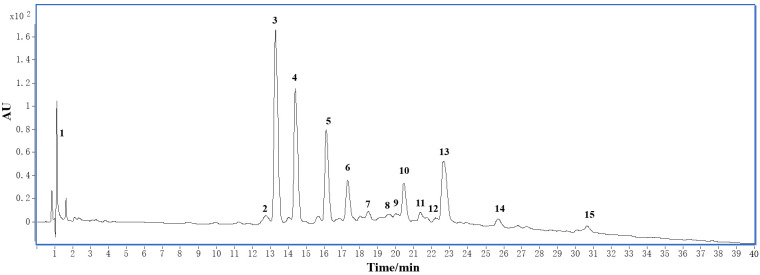
HPLC chromatogram of 70% methanol extract of *C. kwangsiensis*. The numbered peaks (**1**–**15**) correspond to compounds tentatively identified by LC-MS/MS (Table 2): **1**, quinic acid; **2**, **3**, **5**, and **10**, proanthocyanidins B4, B2, C1, and B5, resp.; **4**, (-)-epicatechin; 6, not identified; **7**, rutin; **8**, quercetin-3-*O*-*β*-D-glucopyranoside; **9**, quercetin 3-*O*-*β*-D-galactoside; **11**, kaempferol-3-*O*-rutinoside; **12**, quercetin 3-*O*-*α*-L-arabinoside; **13**, quercetin-3-*O*-*α*-L-rhamnopyranoside; **14**, kaempferol 3-*O*-*α*-L-rhamnoside; **15**, quercetin.

HPLC profiles of *C. kwangsiensis* are shown in Figure 1. From which, fourteen peaks including four proanthocyanidins, nine flavonols and their glycosides, one catechin, and one phenolic acid were identified (Table 2), according to their retention times (*t*_R_), absorbance spectra, quasi-molecular and fragment ions, and HPLC analysis comparing with references, most of which were also isolated in the subsequent chemical isolation from *C. kwangsiensis*.

Peaks **2** (*t*_R_ 12.83 min), **3** (*t*_R_ 13.36 min), and **10** (*t*_R_ 20.43 min) in Figure 1 were thought to be flavan-3-ol dimers. They all gave a prominent precursor [M − H]^−^ ion at *m*/*z* 577. Retro-Diels–Alder (RDA) fission, the most important fragmentation when proanthocyanidin dimers were cleaved, leading to the formation of the fragment ion at *m*/*z* 425. A loss of 18 Da (H_2_O) would occur, resulting in a more stable ion *m*/*z* 407 [18]. The negative ions at *m*/*z* 289 and *m*/*z* 287 found in peaks **2**, **3**, and **10** were formed following quinone methide (QM) cleavage of the interflavan bond, indicating that their upper and terminal units were (epi)catechin. Hence, peaks **2**, **3**, and **10** were supposed to be proanthocyanidin dimers. Peak 5 at *m*/*z* 867 [M + H]^+^ was tentatively assigned as a trimeric B-type PAs consisting of (epi)catechin monomeric units. It generated fragment ions in the positive MS/MS spectrum at *m*/*z* 741, corresponding to the loss of phloroglucinol via heterocyclic ring fission, and at *m*/*z* 715 by RDA fission. The positive ions at *m*/*z* 579 and *m*/*z* 291 likely originate from QM cleavage of the interflavan bond between the upper and middle units. Similarly, QM cleavage of the bond between middle and terminal units produced positive ions at *m*/*z* 289. Fragment ions at *m*/*z* 427 were observed as a result of RDA fragmentation of *m*/*z* 579. Based on a comparison of the retention times with those of the standard samples, peaks **2**, **3**, **5**, and **10** were inferred to be proanthocyanidin B4 (**4**), proanthocyanidin B2 (**3**), proanthocyanidin C1 (**7**), and proanthocyanidin B5 (**5**), respectively, which were subsequently isolated and purified from *C. kwangsiensis*.

Peaks **7**–**9** and **11**–**15** in Figure 1 were believed to correspond to flavonols and their glycosides. Among them, peak **15**, with a quasi-molecular ion at *m*/*z* 301, was identified as quercetin based on its molecular weight and UV absorption characteristics. Peaks **7**, **8**, **9**, **12**, and **13** all produced the same MS/MS ion at *m*/*z* 301, which is consistent with different quercetin glycosides. In the same way, peaks **11** and **14** with fragment ion *m*/*z* 285 were inferred to be kaempferol glycosides. Combined with the comparison with standards, they were identified as shown in Table 2, and some of them were isolated further from *C. kwangsiensis*.

The retention time and quasi-molecular ions of peaks 1 (*t*_R_ 1.38 min; *m*/*z* 191 [M − H]^−^, 215 [M + Na]^+^) and 4 (*t*_R_ 14.51 min; *m*/*z* 289 [M − H]^−^, 291 [M + H]^+^) are identical to those of standard quinic acid and (−)-epicatechin (**8**).

### 3.2. Simultaneous Quantification of Five Main Compositions

According to LC-MS analysis, proanthocyanidin B2 (**3**), proanthocyanidin B5 (**5**), proanthocyanidin C1 (**7**), (−)-epicatechin (**8**), and quercetin-3-*O*-*α*-L-rhamnopyranoside (**9**) are the main components in *C. kwangsiensis* (Figure S1) and were, therefore, selected for quantitative analysis based on their high abundance and the commercial availability of corresponding reference standards. Simultaneous quantification of these five components was performed with the external standard method. Regarding linearity, the correlation coefficients (*R*^2^) of five compounds were all higher than 0.999, obtained in the range of 30–400 µg/mL for **3**, **7**, and **8**, and 12–160 µg/mL for **5** and **9** (Table S2). The estimated LOD and LOQ values of each component were also listed in Table S2. To validate the method, the recovery experiments and intra- and inter-day precision tests were performed, and the results are shown in Table S3. As a result, the proposed method was qualified for the simultaneous determination of five main components in *C. kwangsiensis*. The content of **3**, **5**, **7**, **8**, and **9** in *C. kwangsiensis* was determined to be 4.42 ± 0.023, 0.794 ± 0.034, 2.51 ± 0.016, 1.81 ± 0.013, and 1.44 ± 0.052 mg/g, respectively.

### 3.3. Identification of Compounds ***1**–**19***

**Figure 2:**
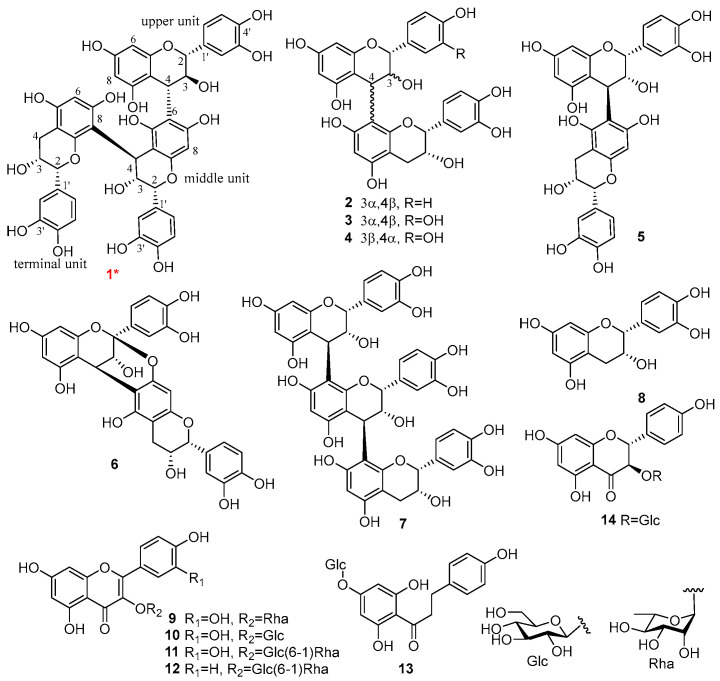
Proanthocyanidins (**1**–**7**) and flavonoids (**8**–**14**) isolated from *C. kwangsiensis*. The red font and * in the figure refer to the new compound.

The EtOAc fraction from the 60% aqueous acetone extract of *C. kwangsiensisv* was processed through repeated CC and semi-preparative HPLC, leading to the isolation of seven proanthocyanidins (**1**–**7**), in which, **1** was a new compound, **2**–**7** were identified as epiafzelechin-(4β → 8)-epicatechin (**2**) [19], proanthocyanidins B2 (**3**) [20], B4 (**4**) [21], B5 (**5**) [22], A6 (**6**) [23], and C1 (**7**) [20], respectively. Furthermore, twelve known phenolic compounds, including one catechin (**8**), six flavonol glycosides (**9**–**14**), and five simple phenols (**15**–**19**), were obtained and identified as (−)-epicatechin (**8**) [24], quercetin-3-*O*-*α*-L-rhamnopyranoside (**9**) [25], quercetin-3-*O*-*β*-D-glucopyranoside (**10**) [26], rutin (**11**) [26], kaempferol-3-*O*-rutinoside (**12**) [26], trilobatin (**13**) [27], (2*R*,3*R*)-(+)-dihydro-kaempferol-3-*O*-*β*-D-glucopyranoside (**14**) [28], (7*S*,8*R*)-guaiacylglycerol (**15**) [29], *E*-*p*-coumaric acid (**16**) [30], lawsorosemarinol (**17**) [31], *p*-hydroxyphenylacetic acid methyl ester (**18**) [32], and *p*-hydroxybenzoic acid (**19**) [33], respectively, through comprehensive spectroscopic analysis and comparison with data from the literature. All the isolates **1**–**19** were first obtained from *C. kwangsiensis*, as depicted in Figure 2 and Figure S2.

**Figure 3:**
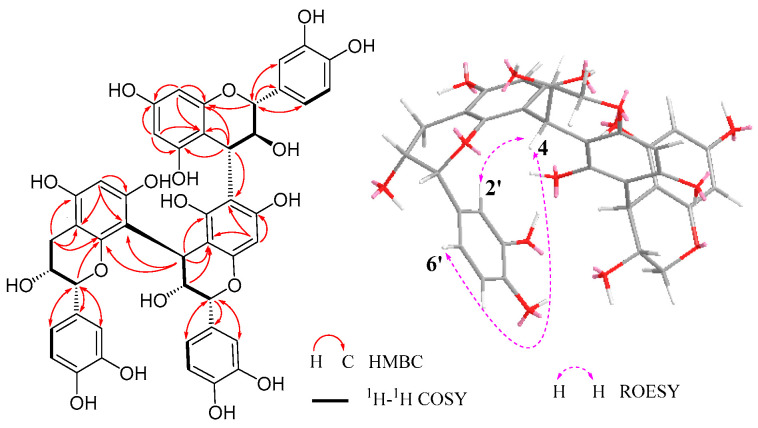
Key 2D correlations (^1^H-^1^H COSY, HMBC, and ROESY) observed for compound **1**.

Compound **1**, ${\left[\alpha \right]}_{D}^{21}$−21.59 (*c* 0.12, MeOH), obtained as orange-red amorphous powder, was assigned the molecular formula C_45_H_38_O_18_ based on the negative HRESIMS analysis (*m*/*z* 865.1980 [M − H]^−^, calcd for C_45_H_37_O_18_: *m*/*z* 865.1985). The IR spectrum displayed a hydroxyl group absorption at 3422 cm^−1^ and characteristic benzene ring stretches at 1612, 1521, and 1446 cm^−1^. The ^1^H NMR data of **1** revealed protons of flavan-3-ol A-rings [*δ*_H_ 5.8–6.0 (Σ 4H)], three sets of ABX signals of B-rings [*δ*_H_ 6.6–7.2 (Σ 9H)], and C-rings [*δ*_H_ 3.9–5.3 (Σ 8H), 2.97 (1H, dd, *J* = 16.8, 4.6 Hz, H-4b), *δ*_H_ 2.82 (1H, dd, *J* = 16.8, 2.5 Hz, H-4a)] (Figure S3). The ^13^C NMR, HSQC, HMBC, and ^1^H-^1^H COSY spectra revealed three sets of *ortho*-dihydroxy-substituted aromatic signals corresponding to three B-rings (Figures S4–S7). This suggested that **1** comprised three catechol-type flavan-3-ol units. Due to the high coupling constant (*J*_2,3_ = 9.6 Hz) of H-2 (*δ*_H_ 4.50)/H-3 (*δ*_H_ 4.70), one of the flavan-3-ol units was deduced to be (+)-catechin. Moreover, the other two sets of H-2 and H-3 at *δ*_H_ 3.9–5.3 showed broad singlet signals, indicating the existence of two (−)-epicatechin units in the compound. Drawing from ^1^H-^1^H COSY and HMBC spectra (Figure 3), the proton and carbon signals of C-rings were attributed as follows: (−)-epicatechin (terminal unit) [*δ*_H_ 2.97 (1H, dd, *J* = 16.8, 4.6 Hz, H-4bT), 2.82 (1H, dd, *J* = 16.8, 2.5 Hz, H-4aT), 4.31 (1H br s, H-3T), 5.00 (1H, s, H-2T); *δ*_C_ 79.9 (C-2T), 67.0 (C-3T), 29.8 (C-4T)], (−)-epicatechin unit (middle unit) [*δ*_H_ 4.73 (1H, d, *J* = 1.6 Hz, H-4M), 3.94 (1H, br s, H-3M), 5.22 (1H, s, H-2M); *δ*_C_ 77.4 (C-2M), 73.3 (C-3M), 37.3 (C-4M)], and (+)-catechin (upper unit) [*δ*_H_ 4.75 (1H, d, *J* = 8.2 Hz, H-4U), 4.70 (1H, m, H-3U), 4.50 (1H, d, *J* = 9.6 Hz, H-2U); *δ*_C_ 83.7 (C-2U), 73.1 (C-3U), 39.2 (C-4U)] (U, M, and L refers to the upper, middle, and lower units, respectively). While the carbon signals in the region of *δ*_C_ 154–158 exhibited some overlap, the assignments for C-5, C-7, and C-9 of each unit were meticulously determined and are detailed in Table 1.

In the HMBC spectrum of **1**, correlations from H-4U to C-6M, as well as from H-4M/H-3M to C-8T, confirmed the interflavan linkages between C-4U and C-6M, and between C-4M and C-8T (Figure 3). Previous study has shown that the ROESY correlations from H-4 to H-2′/H-6′ of the lower unit indicate a 4 → 8-linked proanthocyanidin dimer or trimer [34]. In the ROESY spectrum of **1** (Figure S8), H-4M showed correlations with H-2′T and H-6′T, while H-4U showed no obvious correlation with H-2′M and H-6′M. Thus, **1** was preliminarily deduced as (+)-catechin-(4 → 6)-(−)- epicatechin-(4 → 8)-(−)-epicatechin.

The ROESY crosspeak observed between H-4U and H-2U, together with the large ^3^*J*_2,3_ (9.6 Hz) and ^3^*J*_3,4_ (8.2 Hz) values in the upper unit, determined that the 4 → 6 interflavan bond between the upper unit and the middle unit has an α configuration. However, there is no obvious ROESY correlation between H-4M and H-2M. An all *trans*/*cis* configuration (e.g., 2,3-*cis*-3,4-*cis*) would yield a coupling constant of ^3^*J*_3,4_ ≥ 4 Hz [35,36]. The small ^3^*J*_3,4_ value (<2.0 Hz) of the middle unit suggested a M-4β → T-8 interflavan bond. To confirm the structure of **1**, thiol degradation, which cleaves proanthocyanidin 4 → 8/6 linkages, was performed. Three products were obtained and characterized as (+)-4*α*-(2-hydroxyethyl-sulfanyl)-catechin, (−)-4*β*-(2-hydroxyethyl-sulfanyl)-epicatechin, and (−)-epicatechin, based on comparisons with the literature data [37,38]. Hence, kwangsienin A (**1**) is identified unambiguously as (+)-catechin-(4*α* → 6)-(−)-epicatechin-(4*β* → 8)-(−)-epicatechin.

### 3.4. Antioxidant Activity

Most of the isolates (**1**–**14**) from *C. kwangsiensis* underwent evaluation for their antioxidant activity by DPPH and ABTS^+^ radical scavenging assays. As illustrated in Table 3, almost half of the isolates exhibited superior activities compared to the positive controls (ascorbic acid and trolox, SC_50_ = 17.8 and 188.7 µM, resp.) with SC_50_ values of 5.9–16.8 and 42.5–138.2 µM, respectively. Proanthocyanidins (**1**–**7**) showed significantly greater activities compared to flavonol glycosides (**9**–**14**). This can be attributed not only to the greater number of accessible phenolic hydroxyl groups in the oligomeric proanthocyanidins, which enhances hydrogen-donating capacity, but also to the structural hindrance in flavonol glycosides. Specifically, the *O*-glycosylation at the C-3 position introduces steric bulk that may impede efficient interaction with free radicals [15]. Within the proanthocyanidins, the antioxidant activity order is as follows: trimers (**1**, **7**) > dimers (**2**–**6**) > monomer (**8**). This trend is consistent with the above principle that a higher number of catechol and/or pyrogallol groups in flavan-3-ol units enhances the ability to donate hydrogen atoms and stabilize the resulting phenoxyl radicals. In summary, the key significant differences revealed in Table 3 are as follows: (i) the marked superiority of proanthocyanidins over flavonol glycosides; (ii) a positive correlation between antioxidant potency and the degree of polymerization among low-molecular-weight proanthocyanidins; and (iii) the fact that several proanthocyanidins outperformed standard antioxidant controls.

### 3.5. α-Glucosidase Inhibitory Activity

The *α*-glucosidase inhibitory activities of the isolates (**1**–**14**) were assessed to evaluate the hypoglycemic potential. As indicated in Table 4, a majority of the proanthocyanidins (**1**, **2**, and **4**–**7**) exhibited significant *α*-glucosidase inhibition, with IC_50_ values ranging from 0.91 to 28.8 µM. The activity ranking was as follows: **6** > quercetin > **4** > **5** > **1** > **7** > **2** > **3** > acarbose. Notably, the proanthocyanidin trimers (**1**, **7**) showed weaker *α*-glucosidase inhibitory activity than dimers (**4**–**6**), while flavan-3-ol dimers displayed stronger activity than the monomer (**8**). This inverted “U-shaped” relationship suggests that molecular size and flexibility are critical determinants of inhibitory potency. This finding is consistent with molecular docking studies of proanthocyanidin oligomers, which demonstrated stronger binding affinity (more favourable binding energy) for the dimer compared to the trimer [39]. Our results align with the proposed explanation that dimers achieve an optimal balance between multi-point binding capacity (via phenolic hydroxyls) and molecular accessibility, allowing efficient interaction with the enzyme’s active site. In contrast, the larger and more conformationally constrained trimers may experience greater steric hindrance, slightly compromising their optimal fit into the binding pocket despite their increased number of potential interacting groups.

### 3.6. Anti-Inflammatory Activity

The reduction in NO production serves as a direct indicator of anti-inflammatory activity [40]. Compounds **1**–**3** and **5**–**14** were tested for their ability to inhibit NO production in a murine macrophage cell line. As shown in Table S4, all the tested compounds displayed much weaker NO inhibitory activity than the positive control (L-NMMA) at the concentration of 50 µM. This relative lack of direct, potent NO inhibition at the tested concentration suggests that the primary bioactivity of *C. kwangsiensis* phenolics, under the experimental conditions, may not be mediated through strong suppression of this specific inflammatory pathway. However, it is important to note that inflammation is a complex process. The potent antioxidant activity demonstrated by these compounds could contribute to an indirect anti-inflammatory effect in vivo by mitigating oxidative stress, which is a known trigger and amplifier of inflammatory responses [41]. Further studies using different inflammatory models or higher, non-cytotoxic concentrations would be needed to fully elucidate their immunomodulatory profile.

The present study enriches the phytochemical profile of underutilized species within *Camellia* sect. Thea (Camelliaceae). While several wild relatives, including *C. fangchengensis* [42], *C. taliensis* [15], *C. crassicolumna* var. *multiplex* [43], and *C. sinensis* var. *pubilimba* [44], are also rich in flavan-3-ol derivatives with antioxidant activities, each possesses distinct characteristic compounds. For instance, *C. fangchengensis* features methylene-bridged flavan-3-ol dimers, *C. taliensis* is notable for abundant hydrolyzable tannins, *C. crassicolumna* var. *multiplex* is devoid of caffeine, and *C. sinensis* var. *pubilimba* produces unique C-8 *N*-ethyl-2-pyrrolidinone-substituted flavan-3-ols. In contrast, *C. kwangsiensis* is distinguished by its combination of two key traits: a high content of dimeric/oligomeric proanthocyanidins and a complete absence of caffeine.

The unique “caffeine-free yet proanthocyanidin-rich” profile was further highlighted by direct HPLC comparison with a commercial tea (*C. sinensis* var. *assamica*, Figure S13), which typically contains caffeine and monomeric catechins like (−)-epigallocatechin gallate (EGCG). Therefore, *C. kwangsiensis* occupies a distinct niche, positioning it as a novel botanical resource for developing specialized stimulant-free functional beverages with antioxidant and potential hypoglycemic benefits.

## 4. Conclusions

In summary, 19 compounds, including one new proanthocyanidin (kwangsienin A, **1**), six known proanthocyanidins (**2**–**7**), and 12 known phenols consisting of one catechin (**8**), six flavonol glycosides (**9**–**14**), and five simple phenols (**15**–**19**), were separated and identified from *C. kwangsiensis* growing in Tianlin County, Guangxi Province, P. R. China, for the first time. Among them, **3**, **5,** and **7**–**9** with high yields were characterized as the major components in *C. kwangsiensis* by HPLC and LC-MS analysis. Their content was determined as 4.42 ± 0.023 (**3**), 0.794 ± 0.034 (**5**), 2.51 ± 0.016 (**7**), 1.81 ± 0.013 (**8**), and 1.44 ± 0.052 (**9**) mg/g, respectively. Furthermore, no caffeine, theobromine, or theophylline was detected. Proanthocyanidins (**1**–**7**) demonstrated notable free radical scavenging capability, which was positively related to the count of their constitutive flavan-3-ol units. Most of the proanthocyanidins (**1**, **2**, and **4**–**7**) significantly inhibited *α*-glucosidase with the activity order dimer > trimer > monomer. In addition, the isolates showed weak NO inhibitory activity at a concentration of 50 µM. The findings point to *C. kwangsiensis* as a promising candidate for further development into decaffeinated functional tea products. Future studies focusing on bioavailability, safety, and in vivo efficacy are warranted to substantiate its health-promoting potential. As the main bioactive components, proanthocyanidins serve a key role in the health function of *C. kwangsiensis*.

This study has some limitations that should be considered. The bioactivities are based on standard in vitro assays, which, though suitable for initial screening, have limited physiological predictive power. The modest anti-inflammatory activity suggests this may not be a primary functional pathway. Future work should focus on the following: (i) cellular models under oxidative/hyperglycemic stress; (ii) enzyme kinetics of *α*-glucosidase inhibition; and (iii) in vivo validation of hypoglycemic and antioxidant effects. Our in vitro findings provide a solid foundation for these follow-up studies.

## Figures and Tables

**Table 1:** ^13^C (150 MHz) and ^1^H (600 MHz) NMR spectroscopic data of **1** in CD_3_OD (*δ* in ppm) ^a^.

Position ^b^	Upper Unit		Position ^b^	Middle Unit		Position ^b^	Terminal Unit	
	*δ*_C_	*δ*_H_ (*J*, Hz)		*δ*_C_	*δ*_H_ (*J*, Hz)		*δ*_C_	*δ*_H_ (*J*, Hz)
2	83.7	4.50 d (9.6)	2	77.4	5.22 s	2	79.9	5.00 s
3	73.1	4.70 m	3	73.3	3.94 br s	3	67.0	4.31 br s
4	39.2	4.75 d (8.2)	4	37.3	4.73 d (1.6)	4	29.8	2.97 dd (16.8, 4.6) 2.82 dd (16.8, 2.5)
5	157.2		5	156.7		5	156.8	
6	97.8	5.86 d (2.2)	6	107.9		6	97.8	5.96, s
7	157.6		7	157.2		7	155.8	
8	96.3	5.86 d (2.2)	8	97.7	6.04, s	8	107.6	
9	158.6		9	157.0		9	154.6	
10	107.3		10	100.9		10	100.6	
1′	132.2		1′	132.8		1′	132.2	
2′	116.5	7.04 d (1.9)	2′	115.1	7.00 d (1.7)	2′	115.4	7.12 d (1.8)
3′	146.6		3′	145.8		3′	146.0	
4′	146.3		4′	145.5		4′	145.8	
5′	116.3	6.83 d (8.0)	5′	116.1	6.73 d (8.2)	5′	116.1	6.77 d (8.0)
6′	121.5	6.91 dd (8.0, 1.9)	6′	118.8	6.65 dd (8.2, 1.7)	6′	119.4	6.93 dd (8.0, 1.8)

Note: ^a^ NMR spectra were recorded on a Bruker Ascend-600 spectrometer. ^b^ NMR assignments were based on HSQC, HMBC, ^1^H-^1^H COSY, and ROESY experiments.

**Table 2:** Tentative identification of major compounds in the 70% MeOH extract of *C. kwangsiensis* by HPLC-MS/MS in both negative and positive ion modes ^a^.

Peak	*t*_R_/min	Positive MS	Positive MS^2^	Negative MS	Negative MS^2^	MW ^b^	Compounds ^c^
**1**	1.38	215 [M + Na]^+^		191 [M − H]^−^ 383 [2M − H]^−^		192	quinic acid
**2**	12.83	601 [M + Na]^+^	409, 291, 289	577 [M − H]^−^	425 [RDA]^−^, 407 [425 − H_2_O]^−^, 289 [C/EC]^−^, 287	578	**4**
**3**	13.36	601 [M + Na]^+^	409, 291, 289	577 [M − H]^−^	425 [RDA]^−^, 407 [425 − H_2_O]^−^, 289 [C/EC]^−^, 287	578	**3**
**4**	14.51	291 [M + H]^+^		289 [M − H]^−^	245 [M−H−COO]^−^	290	**8**
**5**	16.11	867 [M + H]^+^	741 [M−phloroglucinol]^+^, 715 [RDA]^+^, 579, 427 [RDA]^+^, 291 [C/EC]^+^, 289, 245	865 [M − H]^−^		866	**7**
**6**	17.22						not identified
**7**	18.57	633 [M + Na]^+^		609 [M − H]^−^	301 [quercetin]^−^	610	**11**
**8**	19.63	487 [M + Na]^+^		463 [M − H]^−^	301 [quercetin]^−^	464	**10**
**9**	20.05	487 [M + Na]^+^	325	463 [M − H]^−^	301 [quercetin]^−^	464	quercetin 3-*O*-*β*-D-galactoside
**10**	20.43	579 [M + H]^+^	409 [427 − H_2_O]^+^, 291, 289, 127	577 [M − H]^−^	425 [RDA]^−^, 289 [C/EC]^−^	578	**5**
**11**	21.53	595 [M + H]^+^	449 [M−rhamnosyl]^+^, 287 [kaempferol]^+^	593 [M − H]^−^	415, 285 [kaempferol]^−^, 227, 185, 133	594	**12**
**12**	22.13			433 [M − H]^−^	301 [quercetin]^−^	434	quercetin3-*O*-*α*-L-ara-binoside
**13**	22.71	471 [M + Na]^+^	325, 87	447 [M − H]^−^	301 [quercetin]^−^, 271, 244, 227, 197, 175, 145	448	**9**
**14**	25.74	455 [M + Na]^+^		431 [M − H]^−^	285 [kaempferol]^−^, 227	432	kaempferol 3-*O*-*α*-L-rhamnoside
**15**	30.76			301 [M − H]^−^		302	quercetin

Note: ^a^ Compounds were tentatively identified by comparing their accurate mass and MS/MS fragmentation profiles with those of authentic standards (when available) and literature data. ^b^ “MW” refers to molecular weight. ^c^ The listed compounds account for the majority of the major peaks in the chromatogram, confirming proanthocyanidins and flavonol glycosides as the dominant phenolic classes.

**Table 3:** DPPH and ABTS^+^ radical scavenging activities of compounds **1**–**14** from *C. kwangsiensis* ^a^.

Sample	SC_50_ (μM) ^a^		Sample	SC_50_ (μM) ^a^	
	DPPH ^b^	ABTS^+ b^		DPPH ^b^	ABTS^+ b^
Ascorbic acid	17.8 ± 0.3	/	**7**	10.4 ± 0.2	50.8 ± 5.1
Trolox	/	188.7 ± 0.1	**8**	30.1 ± 0.2	138.2 ± 4.8
**1**	5.9 ± 0.1	42.5 ± 3.2	**9**	28.2 ± 0.8	245.6 ± 5.1
**2**	16.8 ± 0.1	96.8 ± 2.6	**10**	26.7 ± 2.3	200.3 ± 5.3
**3**	11.3 ± 0.5	53.3 ± 2.8	**11**	38.5 ± 4.5	360.7 ± 4.2
**4**	10.9 ± 0.9	53.4 ± 1.9	**12**	45.7 ± 0.7	884.9 ± 3.5
**5**	11.0 ± 0.1	66.7 ± 0.3	**13**	32.4 ± 3.0	250.3 ± 3.2
**6**	14.9 ±0.1	122.6 ± 8.8	**14**	21.7 ± 1.4	135.1 ± 2.5

Note: ^a^ Values are given as means ± SD (n = 3). ^b^ SC_50_ refers to the concentration that scavenges 50% of DPPH and ABTS^+^ radicals.

**Table 4:** *α*-Glucosidase inhibitory activities of compounds **1**–**14** from *C. kwangsiensis* ^a^.

Sample	IC_50_ (μM) ^b^	Sample	Inhibition Ratio (%) ^c^
quercetin ^f^	5.94 ± 0.2	quercetin ^f^	63.5 ± 0.9 ^d^
acarbose ^f^	223 ± 10	acarbose ^f^	47.2 ± 1.2 ^e^
**1**	19.6 ± 0.4	**3**	20.9 ± 0.6
**2**	28.8 ± 0.8	**8**	53.0 ± 1.9
**4**	10.8 ± 0.3	**9**	41.4 ± 0.7
**5**	17.9 ± 0.5	**10**	38.1 ± 1.0
**6**	0.91 ± 0.10	**11**	7.81 ± 0.50
**7**	20.1 ± 0.4	**12**	18.4 ± 0.6
		**13**	34.0 ± 0.1
		**14**	31.2 ± 3.8

Note: ^a^ Values are shown as means ± SD (n = 3). ^b^ IC_50_ is the concentration corresponding to half-maximal inhibition of *α*-glucosidase; the inhibition ratio of the test compound needs to be greater than 60% at a concentration of 50 µM before further measuring its IC_50_ value. ^c^ The test samples were used at 50 μM. ^d^ Quercetin was present at a concentration of 10 μM. ^e^ Concentration of acarbose was 200 μM. ^f^ Positive control.

## Data Availability

The original contributions presented in this study are included in the article/Supplementary Materials. Further inquiries can be directed to the corresponding author.

## Supplementary Materials

The following supporting information can be downloaded at https://www.mdpi.com/article/10.3390/foods15030442/s1, Figure S1: HPLC chromatogram of standards (a) and 70% MeOH extract (b) of *C. kwangsiensis* (mobile phase: CH_3_CN/H_2_O); Figure S2: Compounds **15**–**19** isolated from *C. kwangsiensis*; Figure S3: ^1^H NMR spectrum of compound **1** in CD_3_OD; Figure S4: ^13^C NMR spectrum of compound **1** in CD_3_OD; Figure S5: HSQC spectrum of compound **1** in CD_3_OD; Figure S6: HMBC spectrum of compound **1** in CD_3_OD; Figure S7: COSY spectrum of compound **1** in CD_3_OD; Figure S8: ROESY spectrum of compound **1** in CD_3_OD; Figure S9: HRESIMS of compound **1**; Figure S10: IR spectrum of compound **1**; Figure S11: CD and UV spectra of compound **1** in MeOH; Figure S12: OR of compound **1** in MeOH; Figure S13: HPLC comparison of 70% MeOH extract of *C. kwangsiensis* and *C. sinensis* var. *assamica* (mobile phase: CH_3_CN/H_2_O, containing 3.4‰ trifluoroacetic acid); Table S1: ^1^H and ^13^C NMR data of products from compound **1** thiolysis; Table S2: Linear dynamic range and estimated LOD and LOQ for compounds **3**, **5**, **7**, **8**, and **9**; Table S3: Results of recovery experiments and intra- and inter-day precision tests; Table S4: Inhibition rate (%) of compounds **1**–**3** and **5**–**14** (50 μM) on NO production.
