# Supplementary material for: Proanthocyanidins from Camellia kwangsiensis with Potent Antioxidant and α-Glucosidase Inhibitory Activity
Source: Foods. 2026 Jan 26;15(3):442. doi: 10.3390/foods15030442 (PMC12896422; doi:10.3390/foods15030442)
Supplement: Supplementary file 1 [file foods-15-00442-s001.zip › foods-4077236-supplementary.pdf]

Supplementary data contents page

Proanthocyanidins from *Camellia kwangsiensis* with potent antioxidant and *α*-glucosidase inhibitory activity

Na Li [1,†], Qin Ni [1,2,†], Min Chen [3], Hong-Tao Zhu [1], Man Zhang [1], Takashi Tanaka [4], and Ying-Jun Zhang [1,3,*]

[1] *State Key Laboratory of Phytochemistry and Plant Resources of West China, Kunming Institute of Botany, Chinese Academy of Sciences, Kunming 650201, China; lina1@mail.kib.ac.cn (N.L.)*
[2] *Yunnan Institute for Food and Drug Control, Kunming 650500, PR China*
[3] *State Key Laboratory of Phytochemistry & Natural Medicines, Kunming Institute of Botany, Chinese Academy of Sciences, Kunming 650201, China*
[4] *Graduate School of Biomedical Sciences, Nagasaki University, 1-14 Bunkyo-machi, Nagasaki 852-8521, Japan; t-tanaka@nagasaki-u.ac.jp*
* *Correspondence: zhangyj@mail.kib.ac.cn; Tel.: +86-871-6522-3235*
† *These authors contributed equally to this work.*

1. Figure S1. HPLC chromatogram of standards (a) and 70% MeOH extract (b) of *C. kwangsiensis* (mobile phase: CH_3CN/H_2O)
2. Figure S2. Compounds **15-19** isolated from *C. kwangsiensis*
3. Figure S3. ^{1}H NMR spectrum of compound **1** in CD_3OD
4. Figure S4. ^{13}C NMR spectrum of compound **1** in CD_3OD
5. Figure S5. HSQC spectrum of compound **1** in CD_3OD
6. Figure S6. HMBC spectrum of compound **1** in CD_3OD
7. Figure S7. COSY spectrum of compound **1** in CD_3OD
8. Figure S8. ROESY spectrum of compound **1** in CD_3OD
9. Figure S9. HRESIMS of compound **1**
10. Figure S10. IR spectrum of compound **1**
11. Figure S11. CD and UV spectra of compound **1** in MeOH
12. Figure S12. OR of compound **1** in MeOH
13. Figure S13. HPLC comparison of 70% MeOH extract of *C. kwangsiensis* and *C. sinensis* var. *assamica* (mobile phase: CH_3CN/H_2O, containing 3.4‰ trifluoroacetic acid) (EC: epicatechin, EGC: epigallocatechin, C: catechin, EGCG: epigallocatechin-3-*O*-gallate, ECG: epicatechin-3-*O*-gallate)
14. Table S1. ^{1}H and ^{13}C NMR data of products from compound **1** thiolysis
15. Table S2. Linear dynamic range and estimated LOD and LOQ for compounds **3**, **5**, **7**, **8** and **9**
16. Table S3. Results of recovery experiments and intra- & inter-day precision tests
17. Table S4. Inhibition rate (%) of compounds **1-3**, **5-14** (50 μM) on NO production

Figure S1. HPLC chromatogram of standards (a) and 70% MeOH extract (b) of *C. kwangsiensis* (mobile phase: CH_3CN/H_2O)

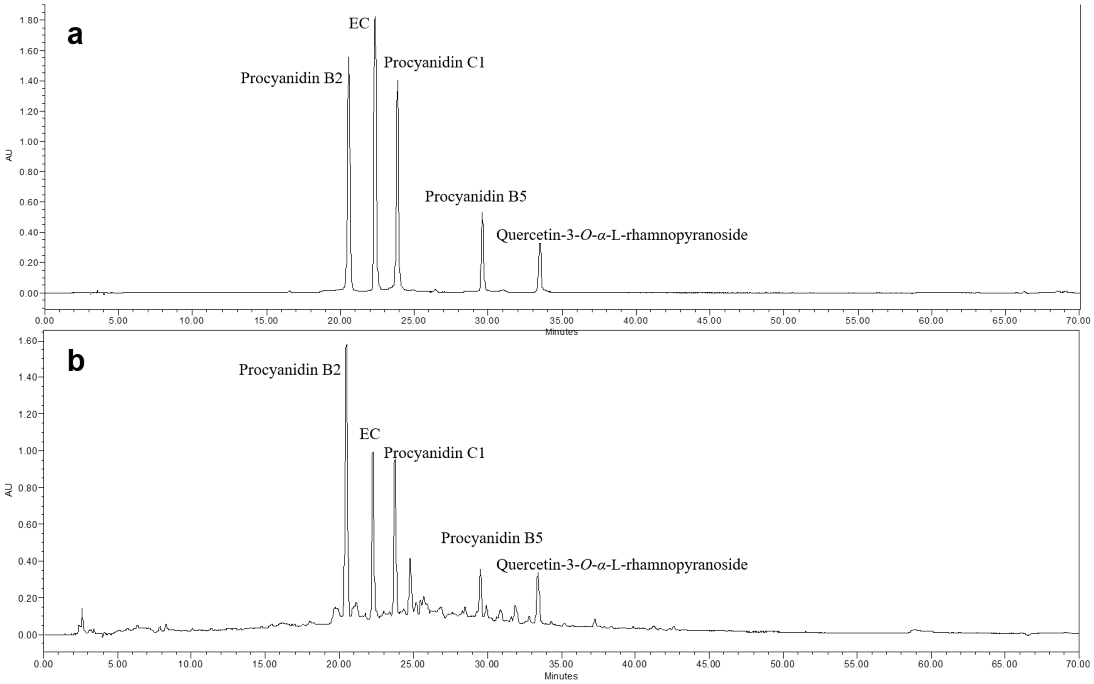

Figure S2. Compounds **15-19** isolated from *C. kwangsiensis*

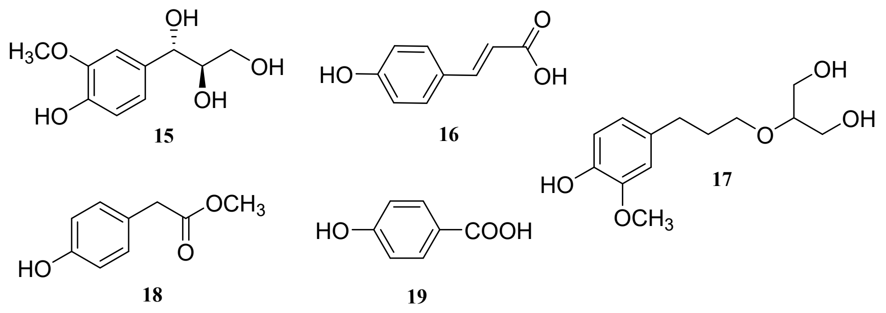

Figure S3. ^{1}H NMR spectrum of compound **1** in CD_3OD

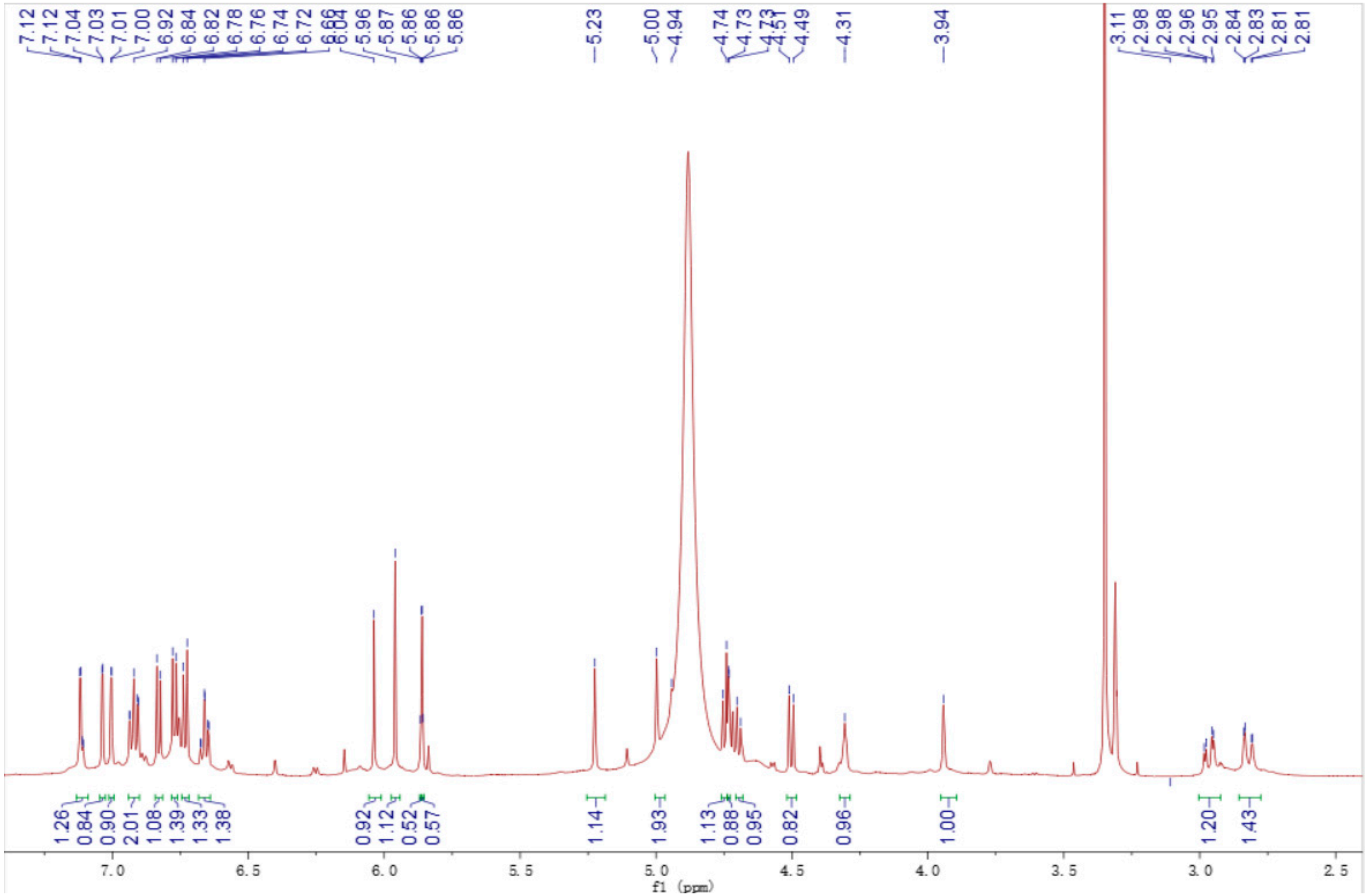

Figure S4. ^{13}C NMR spectrum of compound **1** in CD_3OD

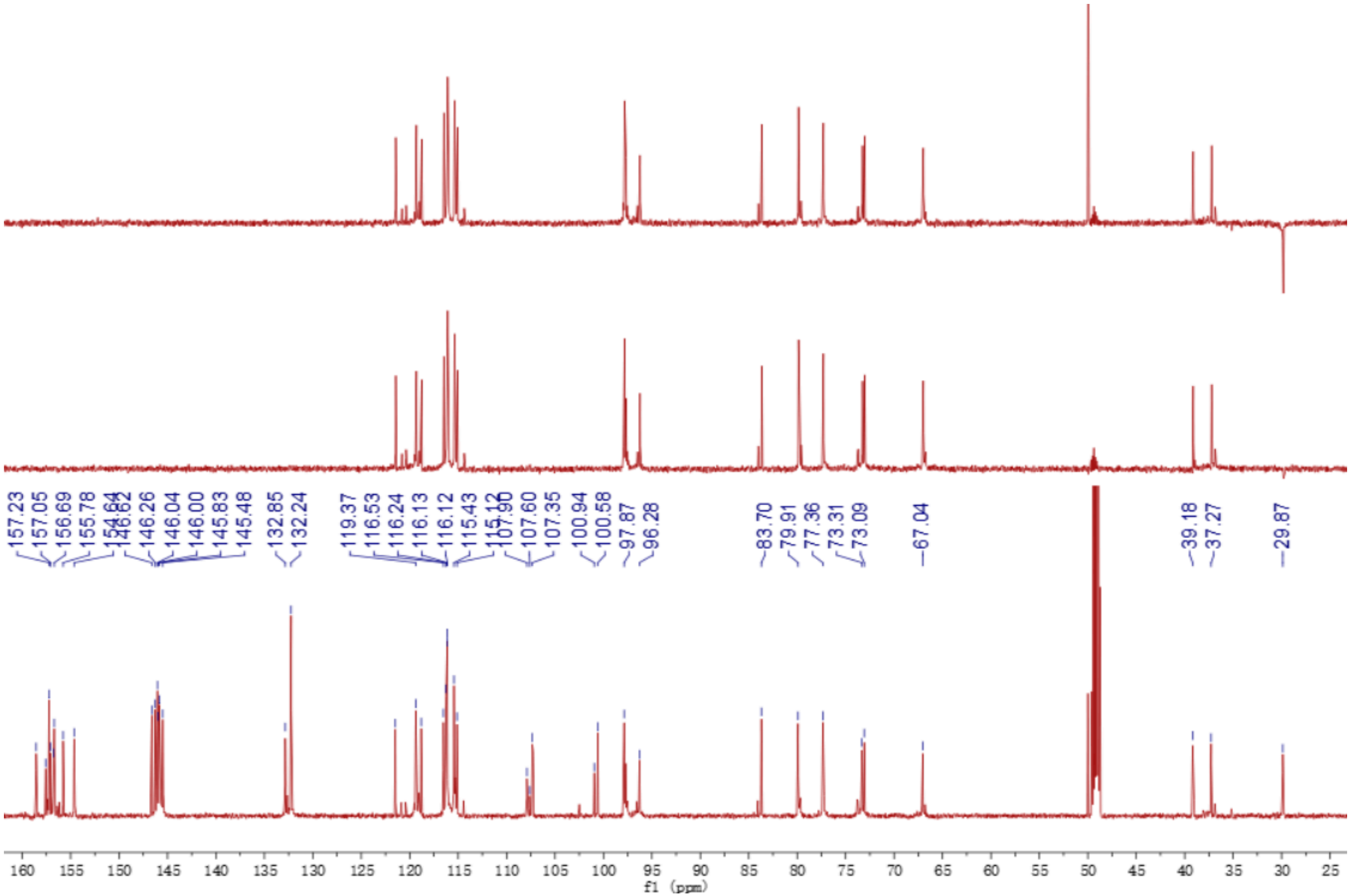

Figure S5. HSQC spectrum of compound **1** in CD_3OD

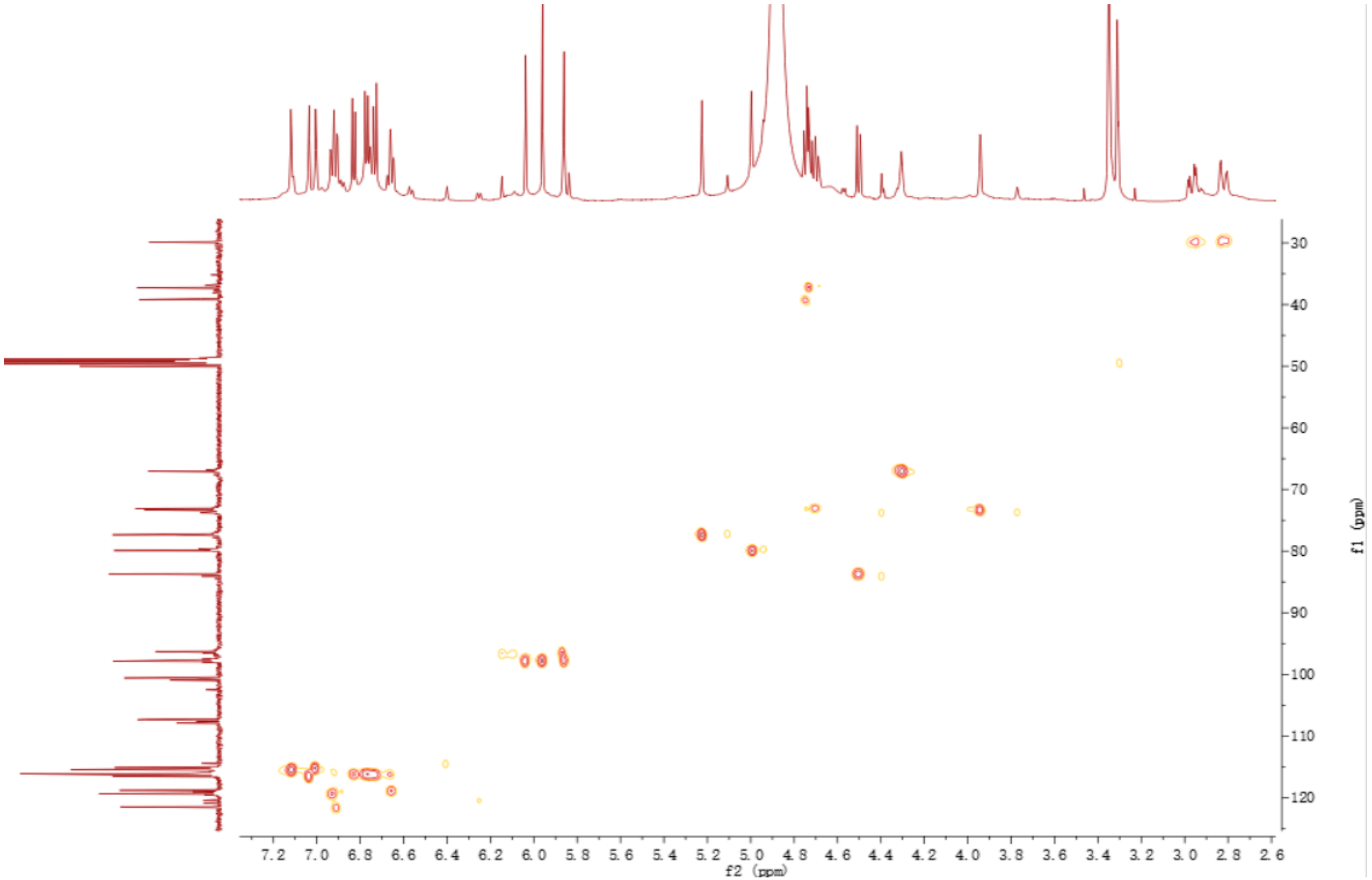

Figure S6. HMBC spectrum of compound **1** in CD_3OD

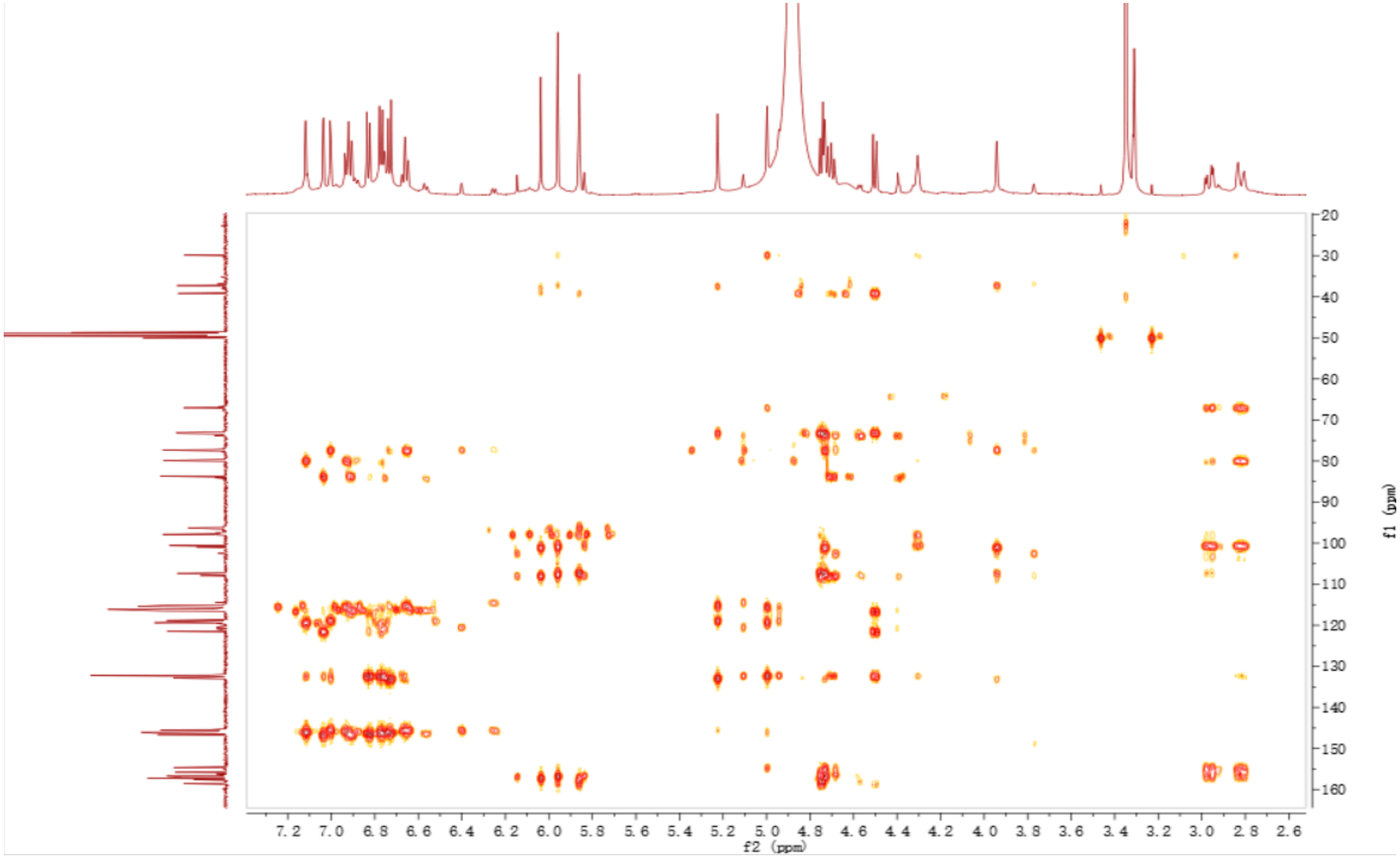

Figure S7. COSY spectrum of compound **1** in CD_3OD

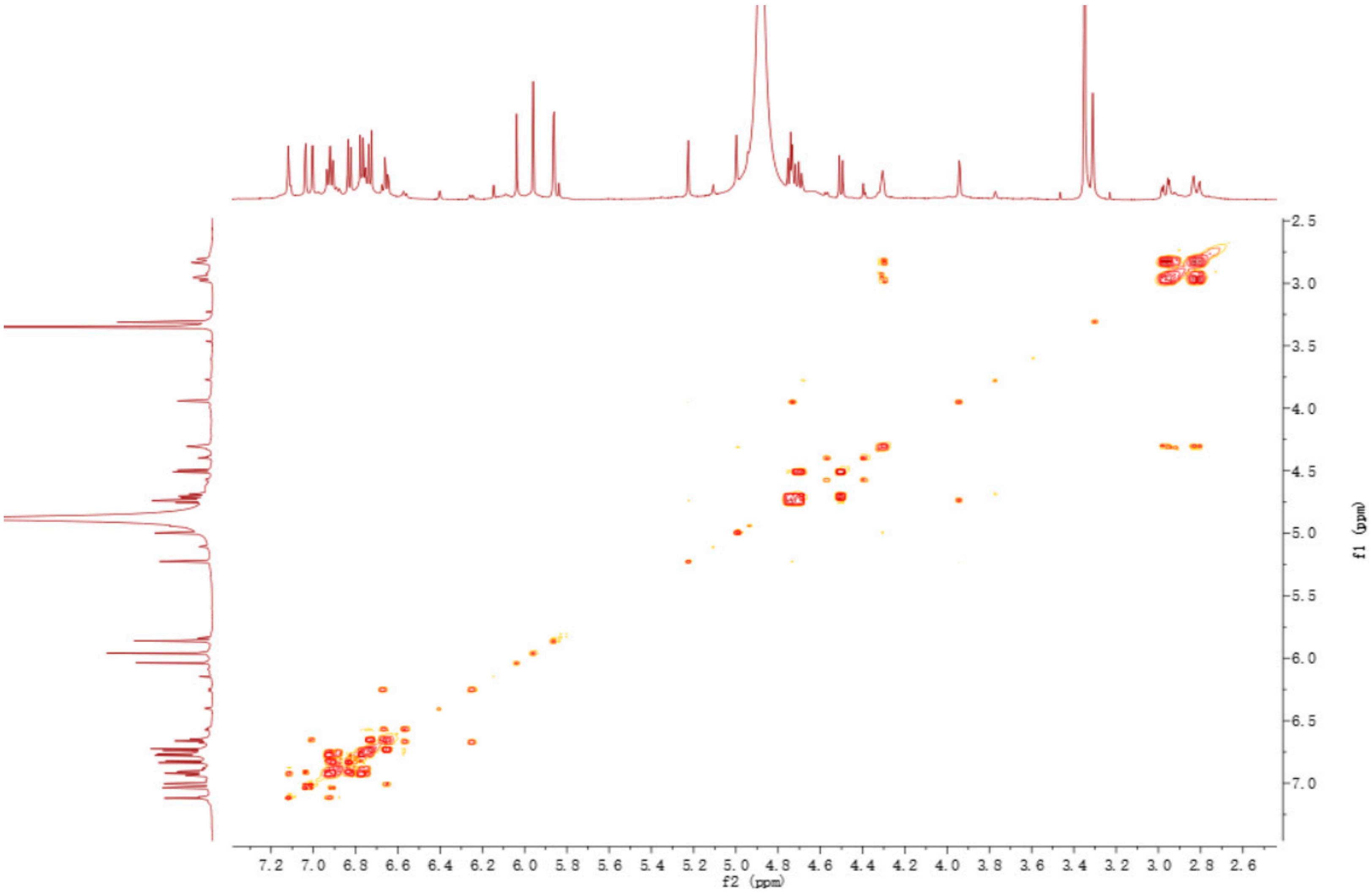

Figure S8. ROESY spectrum of compound **1** in CD_3OD

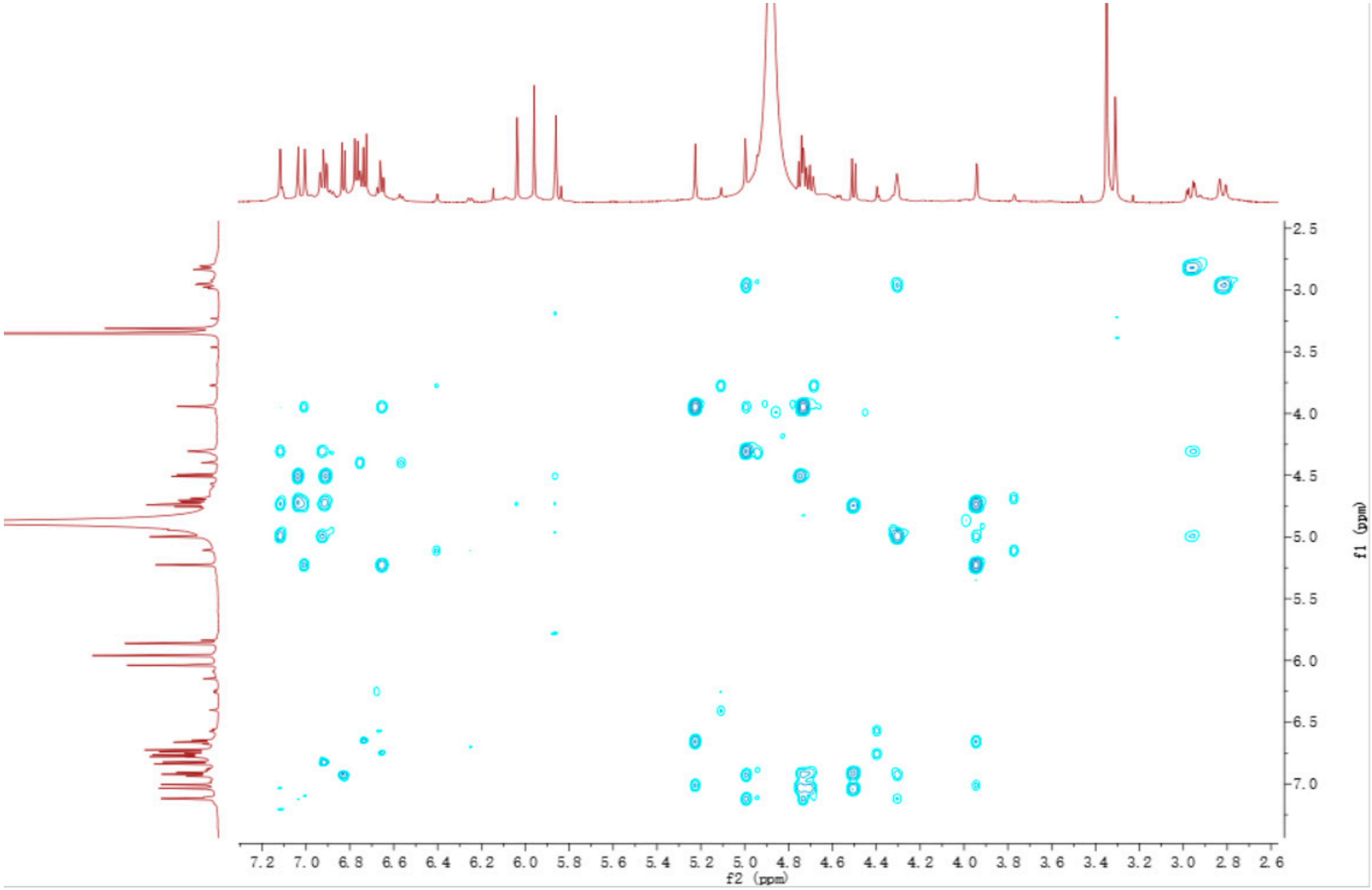

Figure S9. HRESIMS of compound **1**

Formula Predictor Report - yn2-49.lcd

Data File: E:\DATA\2020\0318\yn2-49.lcd

Elmt	Val.	Min	Max	Elmt	Val.	Min	Max	Elmt	Val.	Min	Max	Elmt	Val.	Min	Max	Use Adduct
H	1	10	100	F	1	0	0	S	2	0	0	Br	1	0	0	H
2H	1	0	0	Na	1	0	0	Cl	1	0	0	Pd	2	0	0	
C	4	10	50	Mg	2	0	0	Co	2	0	0	Ag	1	0	0	
N	3	0	10	Si	4	0	0	Cu	2	0	0	I	3	0	0	
O	2	0	30	P	3	0	0	Se	2	0	0					

Error Margin (ppm):	5	DBE Range:	-2.0 - 100.0	Electron Ions:	both
HC Ratio:	unlimited	Apply N Rule:	yes	Use MSn Info:	yes
Max Isotopes:	all	Isotope RI (%):	1.00	Isotope Res:	10000
MSn Iso RI (%):	75.00	MSn Logic Mode:	OR	Max Results:	10

Event#: 2 MS(E-) Ret. Time : 0.333 -> 0.400 Scan# : 52 -> 62

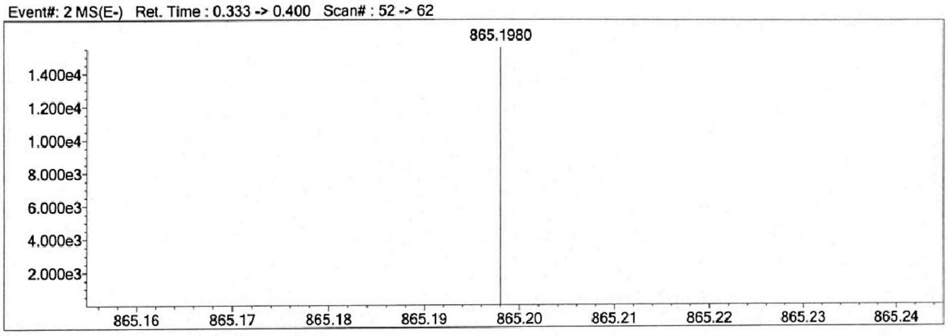

Measured region for 865.1980 m/z

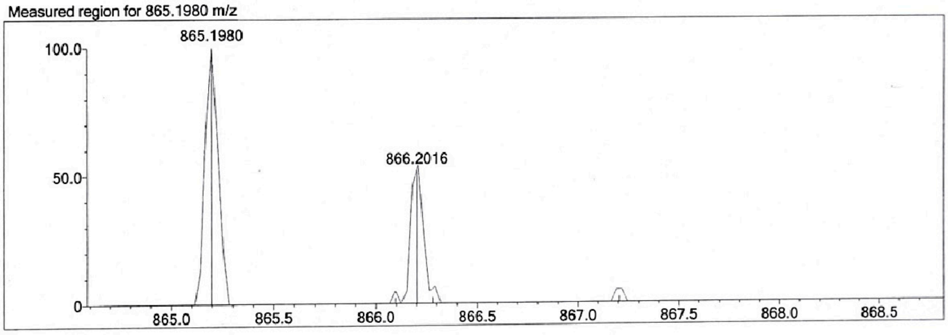

C45 H38 O18 [M-H]- : Predicted region for 865.1985 m/z

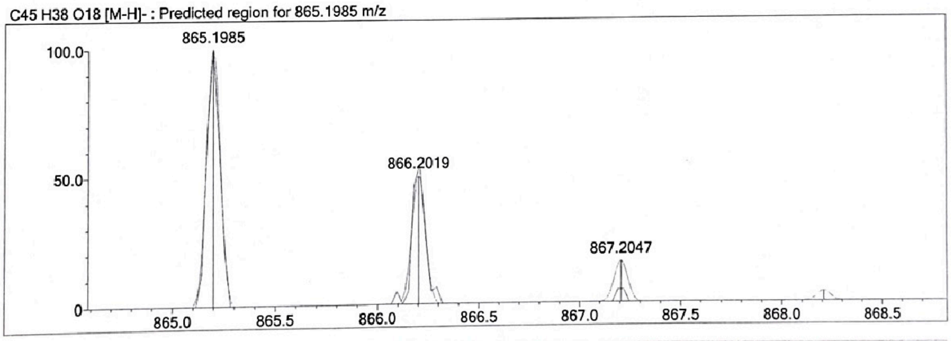

Formula (M)	Ion	Meas. m/z	Pred. m/z	Df. (mDa)	Df. (ppm)	DBE
C45 H38 O18	[M-H]-	865.1980	865.1985	-0.5	-0.58	27.0

Figure S10. IR spectrum of compound **1**

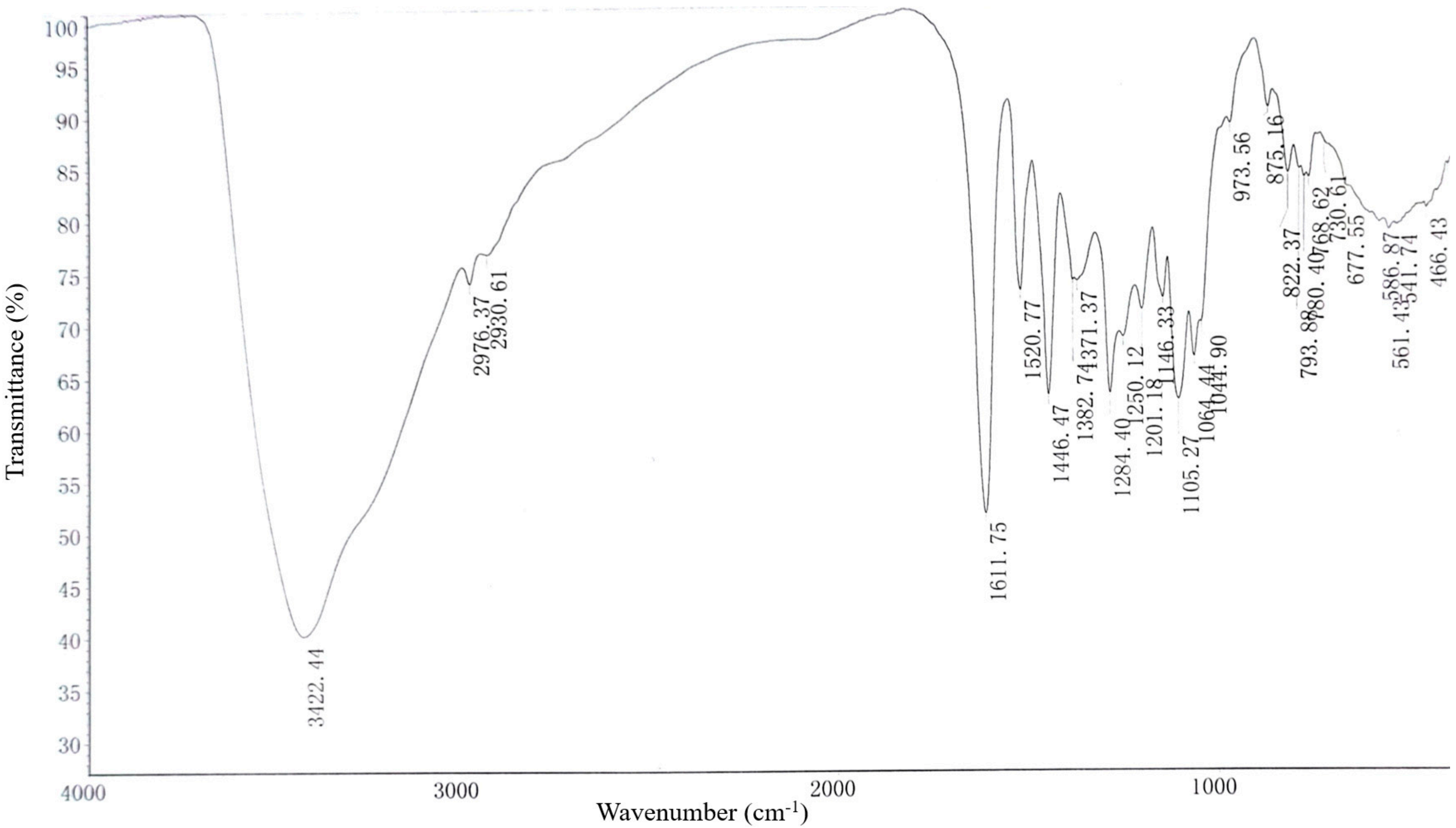

Figure S11. CD and UV spectra of compound **1** in MeOH

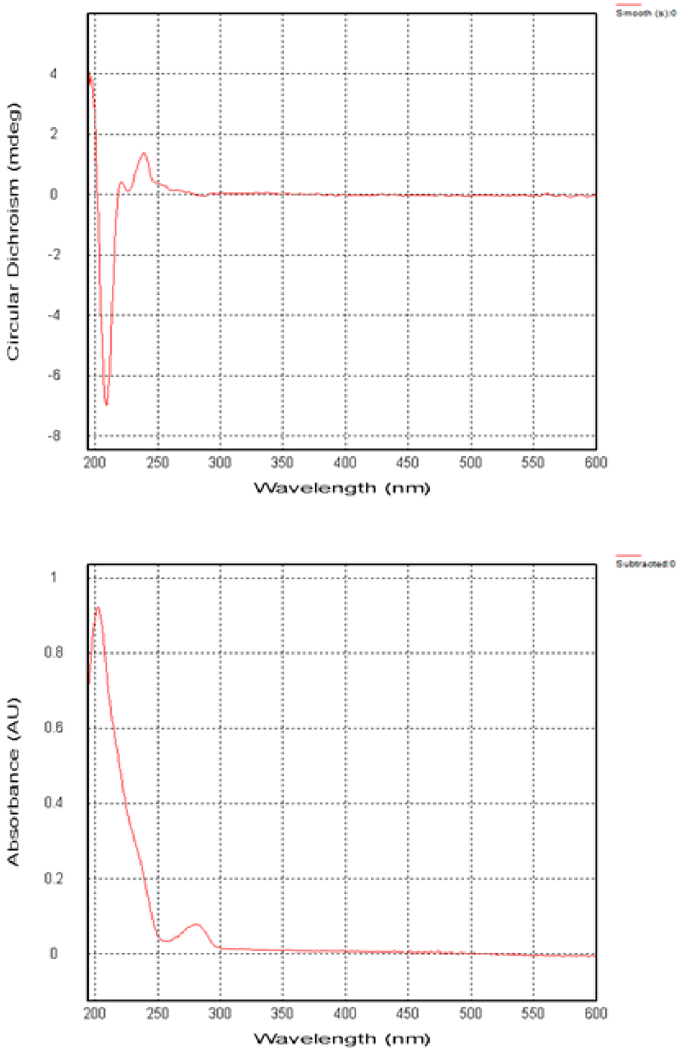

File: YN2-49-1mm(195-600nm)20032002.dsx
ProBinaryX
Attributes :
- Time Stamp :Fri Mar 20 14:27:13 2020

- File ID : {20DBF088-DA02-4512-ACBC-DCE102B81E2D}
- Is CFR Compliant : false
- Original unaltered data

Remarks:
- User: CD
- Date: 2020/03/20
- Instrument: 0547
- DetectorType: LAAPD
- DichOS Calibration Correction Curve: 0547/2
- HV (CDDC channel): 0 v
- Time per point: 1 s
- Description: Sample 1
- Concentration: 0.0413 mg/mL MeOH
- Pathlength: 1 mm
- Temperature: 20℃

Settings:
- HV
- Time-per-point: 1s (25us x 40000)
- SE
- Wavelength: 195nm - 600nm
- Step Size: 1nm
- Bandwidth: 1nm

Figure S12. OR of compound **1** in MeOH

Rudolph Research Analytical

This sample was measured on an Autopol VI, Serial #91058
Manufactured by Rudolph Research Analytical, Hackettstown, NJ, USA.

Measurement Date : Friday, 20-MAR-2020

Set Temperature : OFF

Time Delay : Disabled

Delay between Measurement : Disabled

n	Average	Std.Dev.	% RSD	Maximum	Minimum
5	-21.59	0.22	-1.01	-21.30	-21.87

S.No	Sample ID	Time	Result	Scale	OR °Arc	WLG.nm	Lg.mm	Conc.g/100ml	Temp.
1	YN2-49	01:51:20 PM	-21.87	SR	-0.0269	589	100.00	0.123	21.5
2	YN2-49	01:51:28 PM	-21.63	SR	-0.0266	589	100.00	0.123	21.5
3	YN2-49	01:51:36 PM	-21.30	SR	-0.0262	589	100.00	0.123	21.5
4	YN2-49	01:51:44 PM	-21.46	SR	-0.0264	589	100.00	0.123	21.5
5	YN2-49	01:51:52 PM	-21.71	SR	-0.0267	589	100.00	0.123	21.6

Figure S13. HPLC comparison of 70% MeOH extract of *C. kwangsiensis* (A) and *C. sinensis* var. *assamica* (B) (mobile phase: CH_3CN/H_2O, containing 3.4‰ trifluoroacetic acid) (EC: epicatechin, EGC: epigallocatechin, C: catechin, EGCG: epigallocatechin-3-*O*-gallate, ECG: epicatechin-3-*O*-gallate)

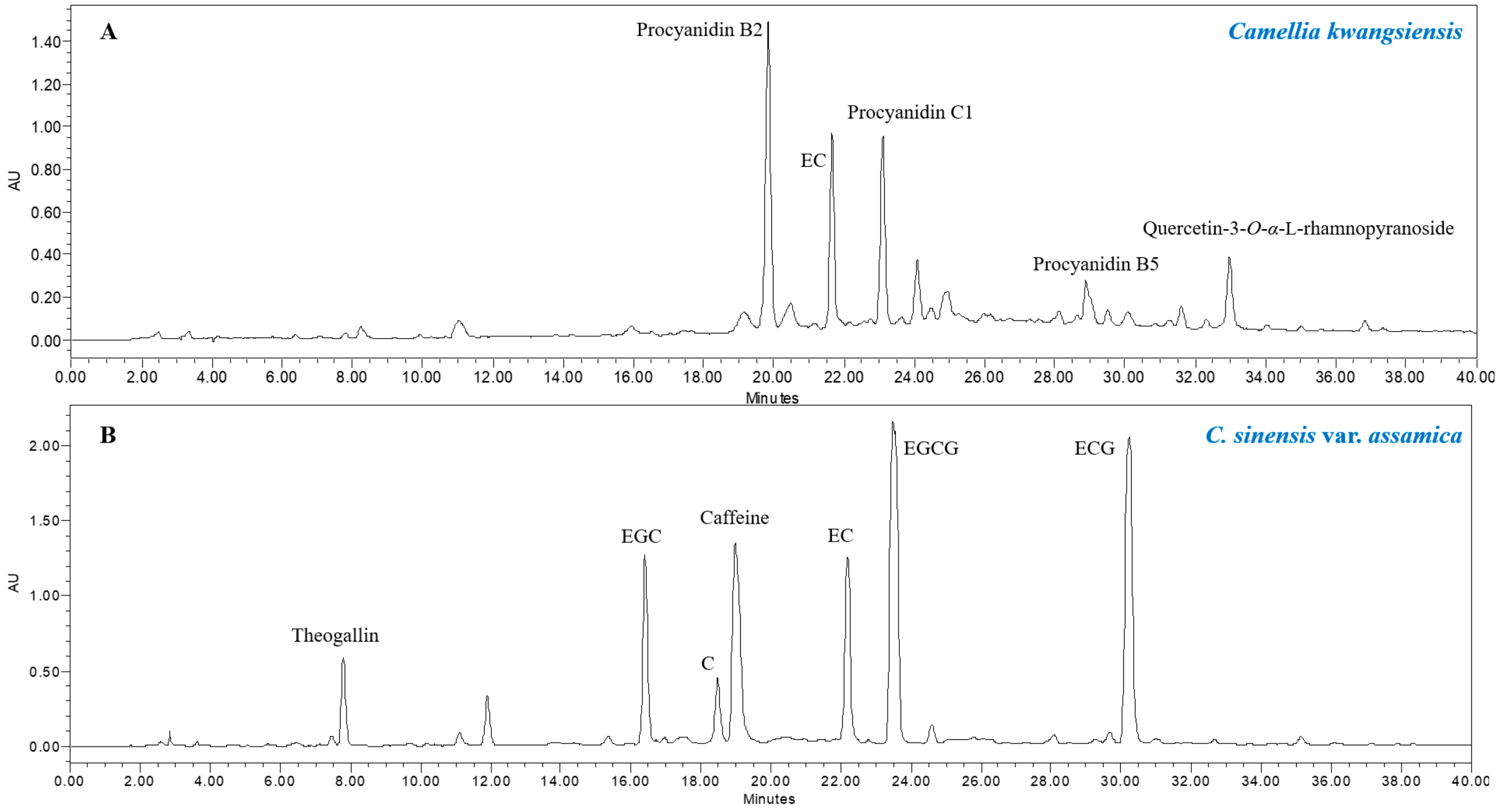

Table S1

^{1}H and ^{13}C NMR data of products from compound **1** thiolysis

No.	C-ME		No.	EC-ME		No.	EC	
	δ_H (*J*, Hz)	δ_C		δ_H (*J*, Hz)	δ_C		δ_H (*J*, Hz)	δ_C
2		79.2	2	5.25 s	75.7	2	4.82 s	80.0
3	4.32 d (4.3)	72.2	3	4.02 d (2.0)	72.6	3	4.18 br s	67.6
4	4.08 m	45.6	4	4.00 d (2.0)	44.2	4	2.73 dd (16.6, 2.4)	29.4
5		156.4	5		157.8	5		157.6
6, 8	5.77 d (2.0)	95.5	6,8	5.91 d (2.0)	95.9	6,8	5.91 d (2.0)	96.0
7		157.8,	7		159.1	7		157.6
9		159.6	9		159.3	9		158.2
10		103.0	10		100.5	10		100.2
1'		131.7	1'		132.2	1'		132.4
2'	6.98 d (2.0)	116.1	2'	7.00 d (2.0)	115.5	2'	6.97 d (2.0)	115.5
3'		146.3	3'		146.0	3'		145.9
4'		146.7	4'		146.2	4'		146.1
5'	6.79 d (8.5)	116.3	5'	6.78 d (8.2)	116.1	5'	6.75 d (2.0)	116.0
6'	6.80 dd (8.5, 1.5)	121.3	6'	6.82 dd (8.2, 2.0)	119.5	6'	6.79 dd (8.3, 2.0)	119.5
SCH_2	2.83-3.03 (2H, m)	37.8	SCH_2	2.64-3.31 (2H, m)	35.7			
OCH_2	3.80 (2H, m)	63.2	OCH_2	3.81-3.85 (2H, m)	63.1			

***Note*:** ME represents hydroxyethylthio ethers, C represents catechin, EC represents epicatechin.

Table S2

Linear dynamic range and estimated LOD and LOQ for compounds **3**, **5**, **7**, **8** and **9**

Compounds	Linear dynamic range (mg/g)	R^2	LOD (μg/g)	LOQ (μg/g)
**3**	0.48 ~ 6.36	0.9993	4.0	12.2
**5**	0.17 ~ 2.88	0.9993	7.2	22.2
**7**	0.47 ~ 6.27	0.9996	11.8	34.3
**8**	0.50 ~ 6.66	0.9992	2.3	7.4
**9**	0.19 ~ 3.14	0.9996	7.6	23.8

Table S3

Results of recovery experiments and intra- & inter-day precision tests.

Compounds	Spiked concentration (µg/mL)	Recovery rate (%)[a]	Concentration for precision tests (µg/mL)	Intraday (µg/mL)[b]	Interday (µg/mL)[b]
**3**	240	91.9 ± 0.99	80	78.7 ± 0.5	82.9 ± 3.0
			300	296 ± 5.2	309 ± 9.9
**5**	120	103.6 ± 0.56	30	28.8 ± 0.5	30.6 ± 1.5
			115	110 ± 2.5	117 ± 3.6
**7**	155	98.5 ± 1.41	75	71.0 ± 1.1	74.4 ± 4.4
			290	282 ± 4.7	288 ± 6.9
**8**	110	99.8 ± 0.42	90	88.0 ± 0.6	93.1 ± 1.8
			320	318 ± 6.3	324 ± 7.0
**9**	100	97.9 ± 2.26	30	28.1 ± 0.3	29.7 ± 0.9
			110	109 ± 2.0	116 ± 3.8

Note: [a]Values represent means ± SD (n = 3). [b] Values represent means ± SD (n = 6).

Table S4

Inhibition rate (%) of compounds **1-3**, **5-14** (50 μM) on NO production[a]

Sample	Inhibition ratio (%)	Sample	Inhibition ratio (%)
L-NMMA[b]	51.9 ± 1.3	**8**	10.4 ±2 .1
**1**	13.7 ± 0.7	**9**	4.30 ± 0.3
**2**	7.63 ± 1.0	**10**	10.7 ± 3.0
**3**	10.0 ± 0.5	**11**	8.32 ± 2.6
**5**	8.10 ± 1.1	**12**	5.95 ± 1.0
**6**	8.41 ± 1.0	**13**	2.24 ± 0.9
**7**	8.07 ± 0.5	**14**	6.45 ± 0.5

Note: [a]Values represent means ± SD (n = 3). [b]Positive control.
